# Case report: A rare case of renal epithelioid angiosarcoma

**DOI:** 10.3389/fonc.2024.1461165

**Published:** 2024-12-02

**Authors:** Jiancheng Zhai, Bangwei Che, Jun Shen, Kangming Cen, Yusui Zhang, Tenxian Li, Dongxin Tang, Kaifa Tang

**Affiliations:** ^1^ Department of Urology and Andrology, The First Affiliated Hospital of Guizhou University of Traditional Chinese Medicine, Guiyang, Guizhou, China; ^2^ Department of Radiology, The First Affiliated Hospital of Guizhou University of Traditional Chinese Medicine, Guiyang, Guizhou, China; ^3^ Department of Pathology, The First Affiliated Hospital of Guizhou University of Traditional Chinese Medicine, Guiyang, Guizhou, China; ^4^ Clinical Medical Research Center, The First Affiliated Hospital of Guizhou University of Traditional Chinese Medicine, Guiyang, Guizhou, China

**Keywords:** scattered calcifications, pathology, renal, epithelioid angiosarcoma, case report

## Abstract

Primary renal epithelioid angiosarcoma (EAS) is extremely rare and carries a poor prognosis. Herein, we present a case of renal EAS in an 81-year-old male patient who complained of hematuria for 1 year. A computerized tomography (CT) scan revealed an occupying lesion at the upper pole of the left kidney, with scattered calcifications, along with retroperitoneal lymph node metastasis and possible lung metastasis. A laparoscopic palliative nephrectomy was performed, and postoperative pathology confirmed a malignant tumor with necrosis in the left kidney. Immunohistochemistry (IHC) revealed positive expression for CD31, CD10, and vimentin, consistent with the diagnosis of EAS. Although EAS is a rare, aggressive, and often misdiagnosed condition, IHC can help confirm its diagnosis, and in our case, the scattered calcifications observed on CT imaging might be helpful in its differential diagnosis.

## Introduction

Angiosarcoma is a rare soft tissue sarcoma originating from endothelial cells, accounting for 1%–2% of all sarcomas (1–3). Epithelioid angiosarcoma (EAS), a morphological subtype, is highly aggressive and has a poor prognosis (3, 4). While EAS is most commonly found in the skin and deep soft tissues, it can also occur in bones, adrenal glands, breasts, and liver (1, 4). Renal EAS is exceptionally rare, with only a few reported cases to date (5–8). The case of primary renal EAS presented here differs in certain aspects from those previously described and could significantly contribute to improving the early diagnosis of this condition.

## Case presentation

**Figure 1:**
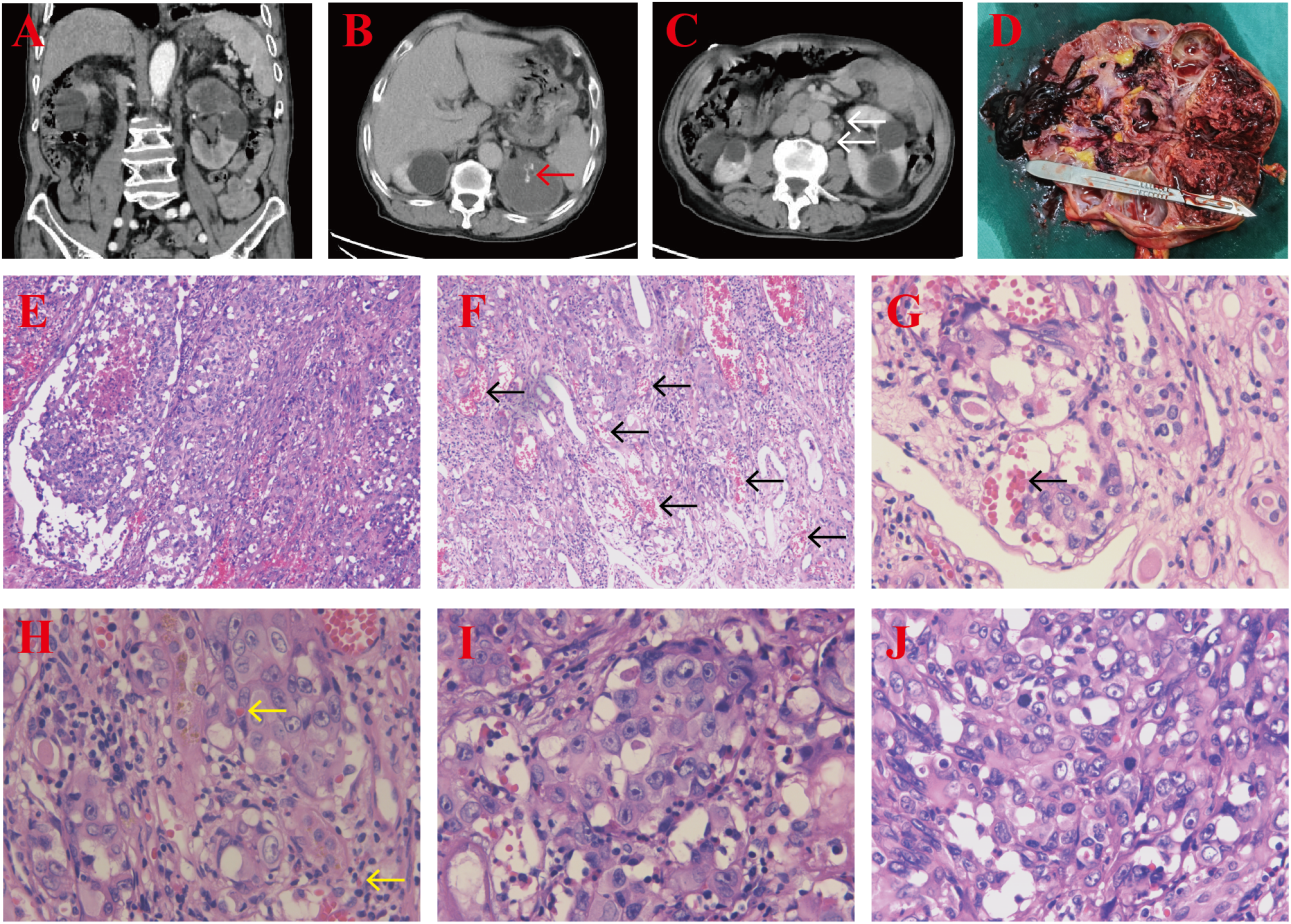
**(A–C)** CT scan showing a quasi-circular soft tissue mass in the upper pole of the left kidney with scattered calcifications (red arrow) and retroperitoneal lymphadenopathy (white arrows). **(D)** The cut section of the tumor revealed a solid, grayish-brown, rough, and brittle lesion, with an unclear corticomedullary junction in the surrounding kidney tissues. **(E, F)** H&E-stained section demonstrating abundant tumor cells arranged in nests and cords, along with irregular vascular spaces (black arrows) (magnification, ×100). **(G, H)** Irregular vascular cavities are rich in red blood cells (black arrow), and individual tumor cells contain red blood cells in their cytoplasm (yellow arrows) (magnification, ×400), H&E staining. **(I, J)** The tumor cells exhibit epithelioid features, with varying nuclear sizes, prominent nucleoli, and visible mitotic figures (magnification, ×400), H&E staining.

**Figure 2:**
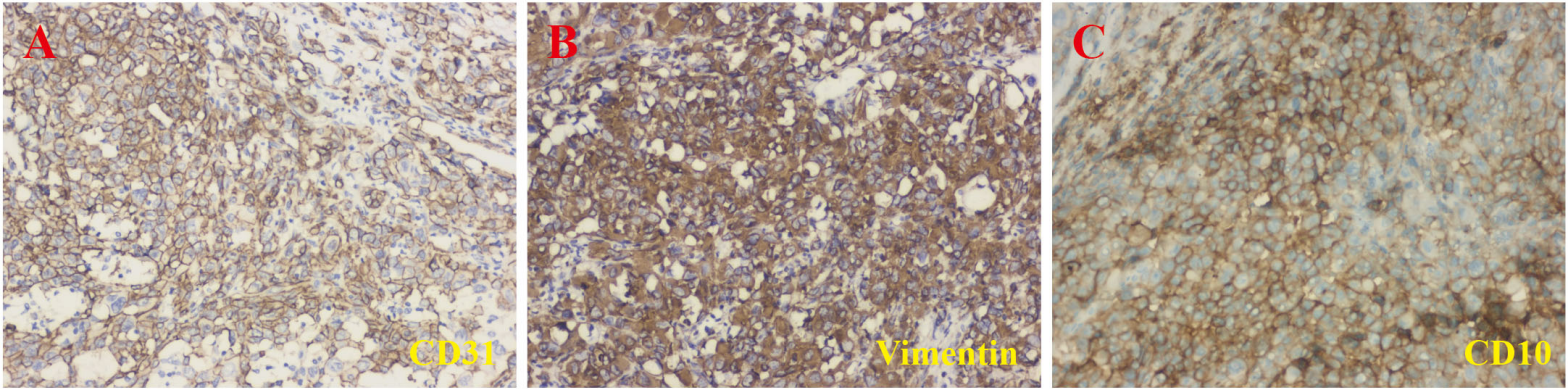
Immunohistochemical staining results. The lesion showed positive staining for CD31 **(A)**, vimentin **(B)**, and CD10 **(C)** (magnification, ×200).

An 81-year-old male patient with a 5-year history of hypertension presented to our hospital with gross hematuria, accompanied by frequent urination and urgency, persisting for 1 year. A computerized tomography (CT) scan revealed a quasi-circular soft tissue mass originating from the upper pole of the left kidney, measuring approximately 72 × 54 mm in diameter. The mass contained scattered calcifications and showed mild enhancement after contrast, along with retroperitoneal lymphadenopathy (**Figures 1A–C**). The CT findings suggested renal cancer, with possible retroperitoneal lymph node and lung metastases. Following a comprehensive evaluation, a laparoscopic palliative nephrectomy was performed. Gross examination revealed a tumor near the upper pole of the left kidney, measuring 60 × 50 × 50 mm. The tumor appeared solid, grayish-brown, rough, and brittle, with unclear corticomedullary junction in the surrounding kidney (**Figure 1D**). Microscopically, at low magnification, the tumor cells were abundant and diffusely arranged in nests and cords, with some areas displaying branched cavities and red blood cell accumulation (**Figures 1E, F**). At higher magnification, irregular vascular cavities are rich in red blood cells, and individual tumor cells contain red blood cells in their cytoplasm (**Figures 1G, H**). The tumor cells varied in size and displayed epithelioid features, with polygonal or spindle shapes, indistinct cell boundaries, abundant eosinophilic cytoplasm, nuclear size variation, prominent nucleoli, and visible mitotic figures (**Figures 1I, J**). Immunohistochemistry (IHC) showed positive staining for CD31, vimentin, and CD10 (**Figure 2**), and negative staining for cytokeratin (CK), CK7, CK19, P504S, PAX-8, CAIX, HMB-45, MELAN-A, S100, SMA, D2-40, and calretinin, leading to the diagnosis of renal EAS. The patient was transferred to the intensive care unit (ICU) postoperatively, but due to complications, including pulmonary infection and his advanced age, he was unable to be weaned from the ventilator. Ultimately, the family decided to discontinue further treatment.

## Discussion

There are seven cases (including this case) of renal EAS reported to date, and the clinical presentation, imaging features, histopathology, and immunohistochemical characteristics are quite similar (5–10). The clinical presentation of EAS often lacks specificity, making preoperative diagnosis challenging. EAS typically occurs in the elderly, and the patient in this case was 81 years old, consistent with previous reported cases (5, 7, 8). Moreover, the clinical symptoms associated with renal EAS may include flank pain, hematuria, and abdominal discomfort, which are common to various renal diseases (11). Herein, the patient presented with gross hematuria, similar to the presentation reported by Liu et al. (7). Other patients with renal EAS may seek medical attention due to pain in the renal area (5). These symptoms are common in other types of renal tumors, making renal EAS diagnosis challenging. As a result, it is difficult for clinicians to make a preliminary differential diagnosis based solely on clinical presentation.

Similarly, the imaging characteristics of EAS often lack specificity, which can lead to challenges in diagnosis. The imaging findings may overlap with those of other renal masses, including renal cell carcinoma and renal epithelioid angiomyolipoma (EAML). For instance, renal EAML can present similarly on imaging studies, often showing a heterogeneous mass with areas of necrosis and fat, which can complicate the differential diagnosis (12). In the present case, the tumor showed mild enhancement after contrast, which could help differentiate it from the significant enhancement typically seen in renal tumors. In a previously reported case of renal EAS, the CT scan revealed a large multilocular lesion in the lower part of the left kidney, characterized by low density and a predominantly fluid-filled content (5). In our case, the CT scan showed a cystic-solid lesion at the upper pole of the left kidney with clear boundaries, low density, and progressive enhancement. These findings suggest that the CT appearance of renal EAS may not exhibit distinctive features. Moreover, in our patient, scattered calcifications were observed within the tumor tissue. Calcifications are often associated with renal cancer, particularly in cases involving osseous metaplasia (13, 14). In a case of bladder angiosarcoma, calcifications within the tumor were identified via ultrasonography, CT, and cystoscopy (15). Similarly, calcifications were also documented in another angiosarcoma case (16). Calcifications in tumors are typically dystrophic, occurring within necrotic regions (15). In one renal EAS case, the CT scan also showed a rounded density shadow, with a ring-like calcification of the wall (10). In our case, necrosis was present in the tumor, and the CT scan showed speckled calcifications in the center of the mass, which may be a specific feature of EAS.

The diagnosis of EAS often relies on pathology and immunohistochemical staining, as clinical symptoms and imaging findings alone cannot distinguish EAS from other renal malignancies. The histopathology differential diagnosis of renal EAS is complex due to its overlapping features with other renal tumors, including clear cell renal cell carcinoma, malignant melanoma, EAML, and malignant mesothelioma (7), and the differential diagnostic basis is shown in **Table 1**. Primary renal EAS could easily mimic epithelial tumor morphologically and immunohistochemically, and may lead to misdiagnosis, but the combined use of endothelial cell markers such as FVIIIRA and CD31 can help confirm its diagnosis (9). Common vascular and epithelial markers, such as CD31, CD34, ERG, and factor VIII, are frequently used to support a definitive diagnosis. However, the positive expression of all these markers is not necessary to confirm the diagnosis (17). Herein, the tumor showed positive expression for CD31, vimentin, and CD10, which led to the final diagnosis of EAS. CD34 positivity has been documented to vary from 40% to 100%, usually staining in regions with abundant vessel formation (18). In the current case, the tumor cells were positive for CD31 but negative for CD34, along with the reported cases (7, 10). As shown in the results in **Table 2**, the positivity rate for CD34 in five renal EAS (two cases were not applicable) cases was 60%. In other reported cases, markers such as CK may also show positive expression (7). However, in our patient, CK was negative. Studies have shown that CK is not a necessary marker for the diagnosis of EAS (19). Moreover, Iacovelli et al. found that in the epithelial-like subtype, the expression of CK and CD31 is more prominent, while the spindle cell subtype is primarily characterized by higher vimentin expression and negative CK expression (20). This pattern appears to align with the immunohistochemical features observed in our patient. Furthermore, CD10 is a specific immunomarker for renal cell carcinoma, but its expression in EAS is rarely reported. In a case of renal EAS described by Singh et al., tumor cells exhibited positive expression of CD10 (8), in line with the positive expression for CD10 in our patient. When CD10 is expressed in EAS, it may complicate the differentiation from renal cell carcinoma with sarcomatoid differentiation. The renal EAS is easily misdiagnosed as epithelioid hemangioendothelioma (EHE), as it could show similar characteristics in immunohistochemical staining, such as being positive for CD31, CD34, and ERG, but the results of Ki-67, WWTR1-CAMTA1, and YAP1-TFE3 might be helpful in differential diagnosis (21, 22). Therefore, when diagnosing EAS based on immunohistochemistry, it is essential to carefully evaluate the relevance of specific markers to arrive at a more accurate diagnosis.

**Table 1:** Differential diagnosis of renal EAS.

Tumor type	Histological examination	Immunohistochemistry	Diagnostic features
Epithelioid angiosarcoma (EAS)	Irregular vascular cavities are rich in red blood cells, and individual tumor cells contain red blood cells in their cytoplasm. Tumor cells varied in size and displayed epithelioid features, with polygonal or spindle shapes, indistinct cell boundaries, abundant eosinophilic cytoplasm, nuclear size variation, prominent nucleoli, and visible mitotic figures.	Positive for CD31, CD34, FVIIIRA, CK, vimentin, CD10, etc.	Individual tumor cells contain red blood cells in their cytoplasm; CD31, ERG, FVIIIRA
Malignant melanoma (MM)	Tumor cells are quite diverse, mainly epithelioid and spindle cells, with small or giant cells showing obvious cell atypia, typical large red nucleoli, easily visible mitoses, and intracytoplasmic visible melanin granules.	Positive for S-100, HMB45, SOX10, MelanA, MART-1, MITF, etc.	Melanin granules; S-100, HMB45, MelanA
Epithelioid malignant mesothelioma (EMM)	The cells are arranged in glandular and papillary shapes, the cells are cuboidal/round, and most of the cells have clear boundaries. The cytoplasm is rich, with red, light red, and purple staining in the cytoplasm. The nucleus is round and oval, with obvious small nucleoli and a small number of mitotic figures.	Positive for calretinin, CK5/6, D2-40, WT-1, CK7, vimentin, EMA, CK18, CK, etc.	Calretinin, D2-40
Clear cell renal cell carcinoma (ccRCC) (poorly differentiated or undifferentiated)	The tumor contains abundant reticular spaces composed of small thin-walled blood vessels. The tumor cells have bright and transparent cytoplasm, round, eosinophilic nuclei, nucleoli of varying sizes, polymorphic nuclei, sarcomatoid structure, and striated muscle differentiation.	Positive for CK, EMA, CAM5.2, Vimentin, CAIX, RCC, CD10, PAX8, PAX2, AMACR, etc.	CAIX, RCC, PAX8
Epithelioid angiomyolipoma (EAML)	Epithelioid tumor cells grow diffusely. The cells are large, oval, and polygonal in shape, with abundant and eosinophilic cytoplasm. The nuclei vary in size, and nucleoli are visible in some cells.	Positive for HMB45, MelanA, cathepsin K, SMA, MSA, Calponin, Vimentin, CD68, Desmin, etc.	HMB45, MelanA, SMA

**Table 2:** Clinical data of seven renal EAS.

No. (Ref)	1 (9)	2 (8)	3 (7)	4 (6)	5 (5)	6 (10)	7	
Age	69	83	75	56	62	78	81	
Gender	Female	Male	Male	Male	Female	Male	Male	
Location	Left kidney	Left kidney	Right kidney	Left kidney	Left kidney	Right kidney	Left kidney	
Clinical presentation	Gross hematuria	Gross hematuria	Gross hematuria	Pain and hematuria	Pain	Gross hematuria	Gross hematuria	
Imaging finding	A left renal mass lesion	A large left renal mass (arrow), extending into perirenal fat	A large mass in the upper-mid portion of the right kidney with mixed density	A nodular mesonephric hypodense lesion characterized by post-contrastographic enhancement	A large multilocular, hypodense predominantly fluid-density lesion along with perinephric fat stranding	A rounded density shadow, with a ring-like calcification of the wall	A cystic-solid lesion with clear boundaries, low density, progressive enhancement, and speckled calcifications in the center of the mass	
Histological examination	Cytoplasm with an epithelial appearance. Intercellular or intracellular microcavities are visible, some containing red blood cells. The nuclei are large, with one to two nucleoli, and mitotic figures are evident.	Pleomorphic epithelioid cells with ample cytoplasm, eccentric nuclei, occasional cytoplasmic hyaline globules, and rare intracytoplasmic lumina.	Pleomorphic epithelioid cells with vesicular nuclei, prominent nucleoli, and eosinophilic cytoplasm that lined irregular vascular spaces.	Marked cellular pleomorphism, enlarged and hyperchromic nuclei, irregular nuclear contour, prominent nucleoli, and frequent mitotic figures were also evident.	Atypical epithelioid cells, eccentric nuclei with coarse chromatin and eosinophilic cytoplasm, vasoformative growth pattern with highly infiltrative vascular channels.	Composed of markedly atypical round or polygonal epithelial-like cells, with mitotic figures easily observed. The cell arrangement shows irregular strands, sheets, and a network of fissure-like structures, with tumor cells covering the edges of the fissures.	The tumor cells varied in size and displayed epithelioid features, with polygonal or spindle shapes, indistinct cell boundaries, abundant eosinophilic cytoplasm, nuclear size variation, prominent nucleoli, and visible mitotic figures.	
Immunohistochemical staining								Possibility
CD31	Positive	Positive	Positive	NA	Positive	Positive	Positive	100%
CD34	NA	Positive	Negative	Positive	Positive	NA	Negative	60%
FVIIIRA	Positive	Positive	Negative	Positive	Positive	Positive	NA	83%
CK	Positive	Negative	Positive	Positive	Positive	Positive	Negative	71%
Vimentin	NA	Positive	Positive	NA	NA	Positive	Positive	100%
CD10	NA	Positive	Negative	Negative	NA	Positive	Positive	60%
S-100	Negative	Negative	NA	Negative	NA	NA	Negative	0%
HMB-45	Negative	Negative	NA	NA	NA	Negative	Negative	0%
Ki-67	NA	NA	30%	40%	NA	50%	40%	NA
Therapy	Operation + chemotherapy	Chemotherapy	Operation + radiation therapy	Operation	Chemotherapy	Operation	Operation	
Follow up	Multiple metastases and death 7 months after surgery	NA	No recurrence was found during 6 months of postoperative follow-up.	The patient came to death a few months later due to a massive hemothorax.	NA	NA	The patient failed to be weaned from the ventilator and the family decided to discontinue further treatment.	

NA, not applicable.

## Conclusion

EAS is a rare disease with a poor prognosis, and its occurrence in the kidney is even more uncommon. Renal EAS lacks characteristic clinical presentations, making the diagnosis largely reliant on pathology and immunohistochemical results. In this case, the presence of calcifications within the tumor tissue identified by CT scan may aid in diagnosing EAS. Nevertheless, further cases are needed for validation.

## Data Availability

The raw data supporting the conclusions of this article will be made available by the authors, without undue reservation.
